# Supplementary figures and images for: L-Arginine Alleviates the Reduction in Photosynthesis and Antioxidant Activity Induced by Drought Stress in Maize Seedlings
Source: Antioxidants (Basel). 2023 Feb 14;12(2):482. doi: 10.3390/antiox12020482 (PMC9952503; doi:10.3390/antiox12020482)

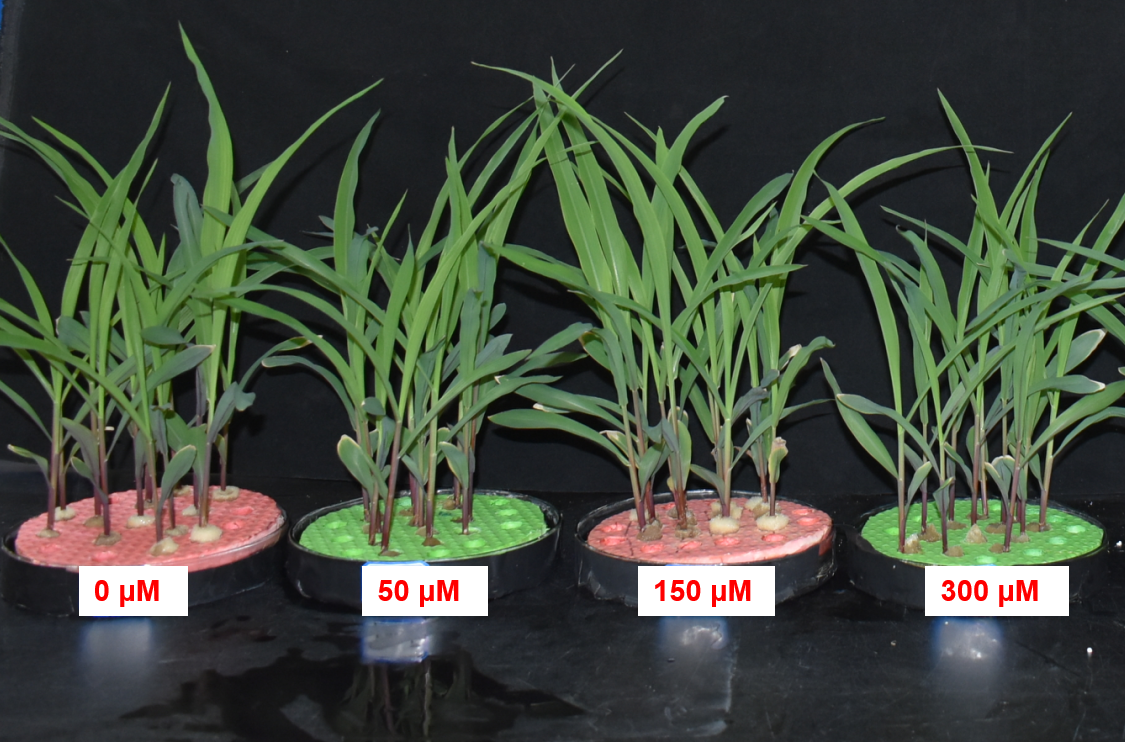

Supplement: Supplementary file 1 [file antioxidants-12-00482-s001.zip › Figure S1..png]

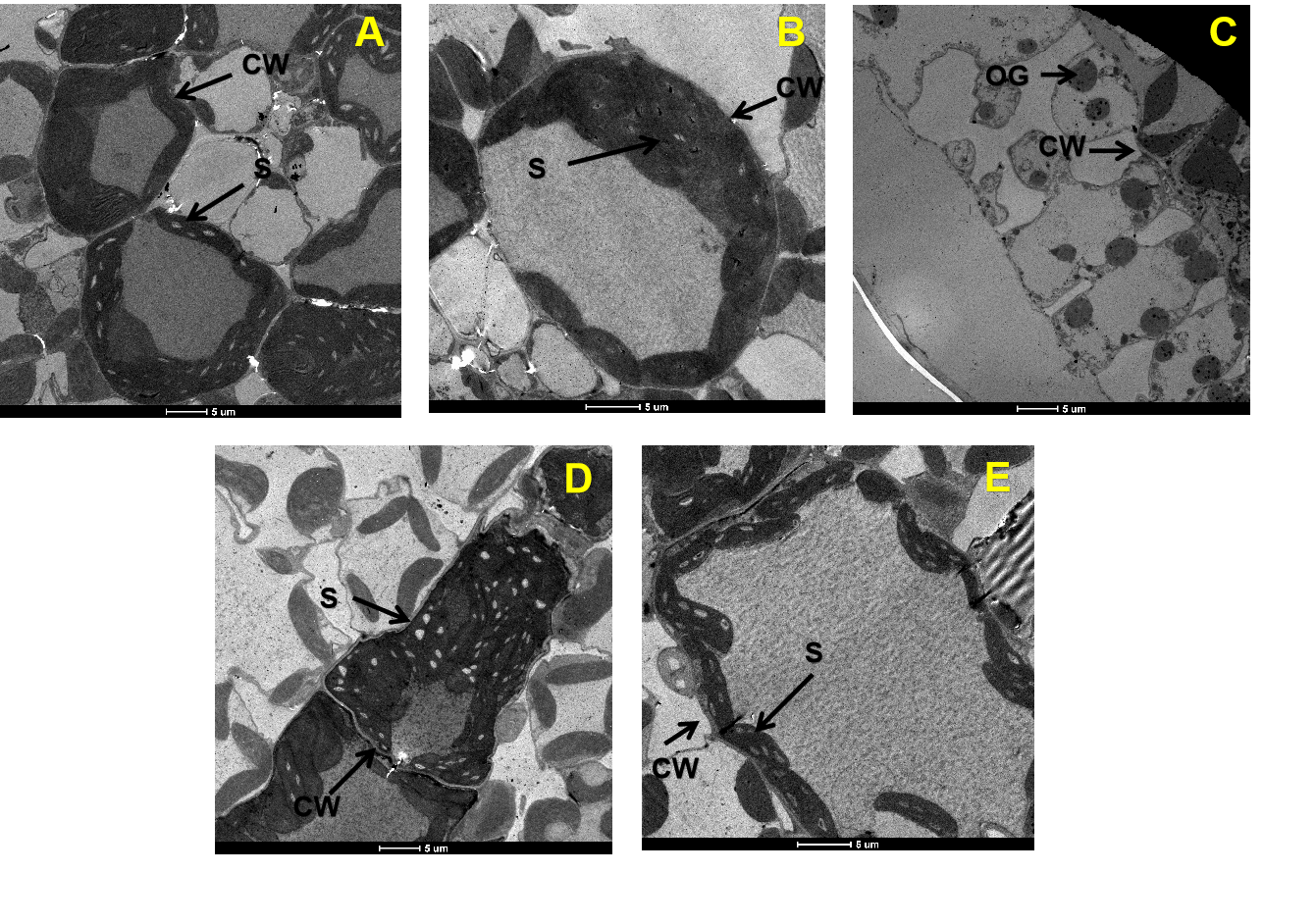

Supplement: Supplementary file 1 [file antioxidants-12-00482-s001.zip › Figure S2..png]
